# Patient safety culture in Chinese nursing homes: a mixed-methods study

**DOI:** 10.3389/fpubh.2026.1821526

**Published:** 2026-09-09

**Authors:** Hui Li, Ying Shi, Jinxia Liu, June Zhang, Zhangan Wang

**Affiliations:** 1 School of Liberal Arts, Guangxi University, Nanning, China; 2 Department of Geriatric Medicine, Shenzhen People’s Hospital, Shenzhen, China; 3 Department of Out Patient Services, The People’s Hospital of Guangxi Zhuang Autonomous Region, Nanning, China; 4 School of Nursing, Sun Yat-Sen University, Guangzhou, China; 5 Department of Health Management, The People’s Hospital of Guangxi Zhuang Autonomous Region, Nanning, China

**Keywords:** adverse events, mixed-methods study, nursing homes, patient safety culture, safety

## Abstract

**Objective:**

To evaluate patient safety culture (PSC) and its associated factors and staff perceptions of adverse event reporting in Chinese nursing homes.

**Design:**

A mixed-methods study based on a secondary analysis of a multisite research program, integrating a 2021–2022 cross-sectional survey with a secondary thematic analysis of focus group interviews.

**Setting and participants:**

Eleven nursing homes in South China, including 674 frontline staff (quantitative phase) and 35 managers, nurses, and caregivers (qualitative phase).

**Methods:**

PSC was assessed using the Nursing Home Survey on Patient Safety Culture. Quantitative data were analyzed using descriptive statistics, univariate analysis, and multiple linear regression. Interview data were analyzed using Braun and Clarke’s thematic analysis.

**Results:**

The overall positive response rate (PRR) for PSC was 86.5%, with lower PRRs for Adherence to Workflow (72.7%) and Staffing (67.0%). PSC was statistically associated with income satisfaction (B = 0.174, *β* = 0.189, *p* = 0.020), job satisfaction (B = 0.176, *β* = 0.208, *p* = 0.046), and years of work experience in nursing home care (B = −0.098, *β* = −0.113, *p* = 0.014). The model explained 35.0% of the variance (*R*^2^ = 0.350, adjusted *R*^2^ = 0.345). Thematic analysis identified four themes: workforce shortages and safety anxiety, non-standardized workflows and interprofessional role conflicts, cultural barriers to adverse event reporting, and staff-driven improvement strategies.

**Conclusions and implications:**

Despite positive overall PSC, structural deficiencies were observed in staffing and adherence to workflow. Cultural factors and organizational pressures may be associated with the underreporting of adverse events. The findings suggest priorities for future intervention development, including resource optimization, workflow standardization, and non-punitive reporting systems. However, these findings require testing in longitudinal or interventional studies.

## 1 Introduction

Aging represents one of the most profound social transformations of the 21st century. By 2050, the global population aged 60 and above is projected to reach 2.1 billion. China, which accounts for one-fifth of the world’s older population and whose older population is growing at an average annual rate of 3%, has one of the most significant aging trends, posing unprecedented challenges to long-term care systems (1). In this context, nursing homes, as core institutions providing continuous and comprehensive care, play an increasingly prominent role in safeguarding the health and well-being of older adults. Nursing home residents commonly have complex health issues, including multiple morbidities, cognitive impairment, and limited activities of daily living. These conditions contribute to the frequent occurrence of adverse events such as falls, unplanned extubations, medication errors, and pressure injuries that reduce the safety and quality of life of older residents (2). Consequently, enhancing patient safety within nursing homes has emerged as a critical public health priority for addressing the challenges of aging and ensuring the well-being of the older population.

Patient safety culture (PSC) is widely recognized as a key non-technical factor associated with safety incidents. It is associated with safety decisions and behavioral norms through the shared values, beliefs, and behavioral patterns of organizational members, and is significantly correlated with nursing quality (3). The theoretical framework of PSC emphasizes internal organizational mechanisms, such as commitment to safety, communication, learning, and improvement, as well as staff perceptions and responses to safety risks. Together, these factors are associated with safety behaviors and serve as vital indicators of an organization’s prioritization of safety. A positive PSC is significantly associated with favorable outcomes, including a lower incidence of adverse events, higher reporting of such events, and improved satisfaction among both staff and residents. PSC is associated with lower rates of specific incidents, such as falls and medication errors, improved timeliness and completeness of nursing care, reduced resident complaints, as well as enhanced service quality and operational efficiency (4). Thus, cultivating and maintaining a positive PSC has become a core strategy for improving patient safety in nursing homes globally.

Despite this global recognition, significant regional disparities and methodological limitations persist in PSC research and practice. Developed countries in Europe and America have actively pursued PSC research in nursing homes since the 1990s, developing assessment tools like the Nursing Home Survey on Patient Safety Culture (NHSOPSC) (5) to measure and improve PSC (6). In contrast, research on PSC in China started relatively later, and existing studies often lack an in-depth critical analysis of contextual explanations within the unique Chinese socio-cultural context. For instance, unique socio-cultural factors like “face culture” (mianzi), a prominent concept in Chinese society that refers to one’s reputation, dignity, and prestige, which is often maintained through social harmony, avoiding public embarrassment or criticism, and obedience to authority, may be associated with the formation and development of PSC, as well as adverse event reporting behaviors (7).

The existing PSC literature in China predominantly focuses on general hospitals, with relatively few systematic and in-depth analyses of nursing homes. Notably, critical discussions of Chinese socio-cultural preferences and taboos are limited (8). When combined with common characteristics of Chinese nursing homes, which tend to prioritize service over management, as well as physical infrastructure and equipment (e.g., nursing beds, rehabilitation devices, safety monitoring systems, and daily living facilities) over culture, this deficiency may exacerbate the difficulty of building a positive PSC. Traditional family care values, face culture (which emphasizes harmony and conflict avoidance), and obedience to authority may weaken staff willingness to report adverse events, limiting the reporting and resolution of problems. As a result, the underlying processes involved in developing PSC in the Chinese context remain insufficiently elucidated (9).

Most PSC studies employ cross-sectional quantitative designs. Although this approach assesses the status and factors associated with PSC, it struggles to reveal the deep-seated reasons for low scores on certain dimensions or why staff are unwilling to report adverse events (6). Our previous multicenter, cross-sectional study of nursing homes across South China revealed a generally low rate of adverse event reporting and a lack of proactive reporting attitudes. Notably, nearly 30% of the frontline staff reported no adverse events at all (10). Thus, there is a need to move beyond solely quantitative research to understand the motivations behind complex behaviors. Mono-methodological approaches have limited a comprehensive understanding of PSC in Chinese nursing homes, hindering the development of effective intervention strategies adapted to the local environment (10–12). Our previous study further found that adverse event management in nursing homes is a multidimensional, complex issue requiring comprehensive consideration at the individual, organizational, and systemic levels, underscoring the necessity for deeper exploration (13). It is particularly necessary to examine non-structural factors, such as organizational culture, staff psychology, and institutional barriers. Existing research has failed to adequately reveal the unique challenges in building PSC and to highlight research limitations (11). Therefore, although previous studies (7, 10) have laid a foundation for understanding PSC, there remains a significant gap in the transition from the macro context to specific research questions within the unique and increasingly important setting of Chinese nursing homes.

This study addressed this research gap by adopting a mixed-methods design to comprehensively assess the status, associated factors, and possible explanatory pathways of PSC in Chinese nursing homes. By integrating quantitative data with qualitative insights, this study aims to reveal the overall characteristics of PSC and penetrate beyond the surface to interpret the complex organizational behavioral logic underlying the weaker dimensions of PSC within the specific Chinese socio-cultural context (14). Particular attention was paid to how unique cultural factors, such as face culture (mianzi), the “Doctrine of the Mean” (Zhong Yong), and high-power distance (which refers to a cultural orientation in which individuals accept and expect that power is distributed unequally within society and subordinates typically do not challenge authority, resulting in a strong hierarchical structure where leaders are perceived as inherently superior and decision-making is centralized) (15, 16), are associated with the formation of PSC and adverse event reporting behaviors. By exploring these possible explanatory pathways, this study aims to provide empirical evidence and propose approaches for improving patient safety management in Chinese nursing homes (7). Specifically, our core research questions are: (i) What is the current status of PSC in Chinese nursing homes? (ii) What factors are associated with the positive response rate (PRR) of PSC in Chinese nursing homes? and (iii) What are the possible explanatory pathways associated with the weaker dimensions of PSC within the socio-cultural context of China?

This study is a secondary analysis of data from a multisite research program on safety management in Chinese nursing homes. The quantitative data derive from the program’s 2021–2022 cross-sectional survey wave; the present analysis focuses on the subsample with complete Patient Safety Culture (PSC) data (*N* = 674) and, for the first time, treats PSC as the outcome variable, whereas our previous publications from the same wave examined adverse event incidence and reporting attitudes (10, 12). The qualitative component is a secondary thematic analysis of the program’s focus-group corpus (35 participants), which was previously analyzed to examine staff experiences of adverse event management (13); the present re-analysis is oriented by the PSC framework and addresses a distinct research question. No findings, tables, or analytical results are duplicated across these publications.

## 2 Methods

### 2.1 Overall research design and ethics statement

This study employed a mixed-methods design (17) to comprehensively assess the current status of PSC in nursing homes across South China and examine the factors associated with PSC scores. The study used two complementary data sources from the same multisite research program (quantitative surveys and qualitative focus group interviews) to evaluate the status of various PSC dimensions and their associated factors, identify areas of weakness, and explore possible contextual explanations for these findings.

The original data collection was approved by the Medical Ethics Committee of the Sixth Affiliated Hospital of Sun Yat-sen University, Guangzhou, China and the management boards of all participating nursing homes (Ref No. L2021101). This secondary analysis was conducted within the scope of the original ethical approval and informed consent. Prior to data collection, all participants were fully briefed on the study’s purpose, content, procedures, and potential risks and benefits, with emphasis placed on the voluntary nature of participation, anonymity, and their right to withdraw at any time. Written informed consent was obtained from all participants. Participant privacy and personal information were strictly protected throughout the study, and all data were anonymized.

#### 2.1.1 Quantitative phase

We analyzed data from a cross-sectional survey of frontline staff conducted within the research program from 11 nursing homes in South China using the Chinese version of the NHSOPSC. The objective of this phase was to quantify PSC dimension scores, the PRR, and factors associated with PSC scores (6, 18).

The sample size was determined based on statistical power, the expected effect size, and the scale structure complexity with reference to previous studies (10, 18). We conducted an *a priori* power analysis using G*Power software (linear multiple regression: fixed model, *R*^2^ deviation from zero) (19) with 80% statistical power (power = 0.80), a 0.05 significance level (*α* = 0.05), five predictors in the multiple linear regression model, and a small expected effect size (*f*^2^ = 0.02); the estimated minimum required sample size was 647 cases.

#### 2.1.2 Qualitative phase

The present qualitative analysis was oriented by the weak PSC dimensions identified in the quantitative analysis. The interview data were drawn from the research program’s focus-group corpus and were re-analyzed thematically to address the PSC-specific research question. Interviews aimed to explore organizational behavioral patterns, authentic staff experiences, and the perceived reasons underlying these weaknesses to provide contextual explanations for the quantitative findings.

This mixed-methods design was selected for its ability to effectively synergize the generalizability of quantitative research with the contextual depth of qualitative inquiry to offer a comprehensive understanding of complex phenomena (20). This integrated approach addresses the complexities inherent in PSC research in nursing homes and mitigates the potential lack of explanatory depth associated with mono-methodological approaches, thereby providing a more informed basis for PSC improvement (21).

### 2.2 Participants and sampling methods

#### 2.2.1 Quantitative phase

This study utilized convenience sampling. A broad representation of nursing homes across South China (e.g., government-operated and privately-operated, for-profit, and non-profit) was included to minimize bias and enhance sample diversity and external validity (22). Ultimately, frontline staff from the 11 selected nursing homes in South China were included as participants. The inclusion criteria were: (1) frontline staff with ≥ 1 year of work experience in nursing home care, which spanned various roles, including physicians, registered nurses (RNs), technicians, rehabilitation specialists, caregivers (staff who care for older residents living in nursing homes), and social workers, and (2) individuals who provided informed consent. The exclusion criteria were: (1) individuals unable to independently read and comprehend the questionnaire or use a smartphone due to language barriers or literacy levels, and (2) staff members absent from their posts for > 2 weeks during the survey period.

The quantitative sample was composed predominantly of caregivers (*n* = 567, 84.1%), with licensed professional staff representing a minority: RNs (*n* = 45, 6.7%), physicians (n = 21, 3.1%), technicians/rehabilitation specialists (*n* = 30, 4.5%), and social workers (*n* = 11, 1.6%). Because caregivers constituted the majority of the sample, the quantitative findings should be interpreted as primarily reflecting caregiver perspectives rather than balanced multidisciplinary staff perspectives. All job roles were pooled in the regression analysis because the primary objective was to examine facility-level and individual-level factors associated with PSC across the entire frontline workforce; however, role-specific differences in PSC perceptions, reporting behavior, and power dynamics may exist and represent a limitation of the pooled approach. Further stratified analyses by job role were not conducted due to insufficient sample sizes in the non-caregiver categories.

#### 2.2.2 Qualitative phase

Lived experiences, underlying perceived reasons, and suggestions for improvement on the weaker dimensions of PSC identified in the quantitative phase were explored using a maximum variation purposive sampling strategy to maximize diversity among the participants. This strategy aimed to capture a broad spectrum of perceptions and experiences regarding PSC across different roles, years of service, and facility operation modes (one government-operated and one privately-operated nursing home) (23). Informed by the PSC dimensions with lower scores in the quantitative results and factors such as job role, tenure, and facility ownership, three core roles were selected as the primary participants for focus group interviews: managers, RNs, and caregivers. This selection process was grounded in the logic of mixed-methods theory, which mandates identifying areas of weakness through quantitative data and subsequently probing the complexity and nuances of these areas through qualitative inquiry.

The participants were recruited from the pool of quantitative survey participants and stratified by their job title (i.e., groups for caregivers, RNs, and managers). The inclusion criteria for qualitative interviews were: (1) ≥ 1 year of experience in nursing home care as a frontline caregiver, RN, or manager; and (2) providing informed consent to participate. We excluded staff who were absent from their posts for > 2 weeks during the survey period. Participants were recruited through (1) recruitment posters within the nursing homes outlining the study’s purpose and participation requirements, and (2) face-to-face briefings by researchers explaining the study and its aims. Recruitment continued until data saturation was reached, (see Section 2.4.2 for the saturation criterion). During recruitment, one staff member declined to participate due to scheduling conflicts.

A total of six focus group interviews were conducted: two with caregivers (total *n* = 12), two with RNs (total *n* = 12), and two with managers (total *n* = 11). Managers, RNs, and caregivers were interviewed in separate groups to reduce power dynamics and encourage open expression, and managers were not present during caregiver or nurse interviews. The interviewer emphasized their role as an independent researcher (not an employee or evaluator of the nursing homes), assured participants that their responses would not be shared with management, and facilitated the discussion using neutral, non-judgmental language to mitigate potential power dynamics. All participants were seated in a hollow square arrangement to promote egalitarian participation, and the interviewer explicitly encouraged junior staff to share their views.

### 2.3 Data collection

#### 2.3.1 Quantitative measures

Quantitative data collection utilized a general demographic questionnaire and the Chinese version of the NHSOPSC (18). Originally developed by the Agency for Healthcare Research and Quality (6), the NHSOPSC has demonstrated acceptable psychometric properties in Chinese contexts, and its full Chinese version has been validated in previous studies (18). It comprises 23 items covering six core dimensions: (1) Organizational Learning and Continuous Improvement (six items), (2) Handoff and Communication, originally called handoffs and information exchange (five items), (3) Management Support for Safety (four items), (4) Teamwork (two items), (5) Adherence to Workflow (two items), and (6) Staffing (four items). Items were scored using a 5-point Likert scale (1 = strongly disagree, 5 = strongly agree). Three items are negatively worded and were reverse-coded before dimension scoring, in accordance with the scoring instructions of the validated Chinese version (18).

The overall Cronbach’s α coefficient for the Chinese version of the NHSOPSC was reported to be 0.884 in prior validation studies, with coefficients for individual dimensions ranging from 0.737 to 0.866 (Organizational Learning: 0.866, Handoff and Communication: 0.843, Management Support: 0.822, Teamwork: 0.814, Adherence to Workflow: 0.779, and Staffing: 0.737), and the test–retest reliability was 0.904 (18). In the present study, the analysis of the formal survey data yielded a Cronbach’s α of 0.918 for the total scale, with dimension-specific coefficients ranging from 0.584 to 0.960 (Organizational Learning: 0.960, Handoff and Communication: 0.897, Management Support: 0.937, Teamwork: 0.921, Adherence to Workflow: 0.905, and Staffing: 0.584). The lower α for the Staffing dimension is attributable to its small number of items with heterogeneous content (staffing adequacy across shifts versus staff turnover). The remaining dimensions showed good internal consistency.

The total PSC score was calculated as the mean of all responses to the 23 items after reverse-coding. This approach is consistent with prior NHSOPSC studies (6, 18) and reflects the theoretical conceptualization of PSC as a global organizational climate construct. While the six dimensions are conceptually distinct, the total score provides a summary indicator of the overall PSC climate. We acknowledge that reporting dimension-specific scores with the total score provides more nuanced information.

We calculated the PRR and mean scores to analyze dimensional and item data relating to PSC in nursing homes according to previous studies utilizing the NHSOPSC. The PRR was calculated as the percentage of respondents who responded positively to an item — “strongly agree” or “agree” for positively worded items, and “strongly disagree” or “disagree” for the three reverse-coded items. Dimensions or items for which the PRR was > 75% were classified as areas of strength, while those for which the PRR was < 50% were classified as areas requiring improvement, thereby providing an empirical basis for selecting focal points for subsequent qualitative inquiry. PRR thresholds are used as heuristic benchmarks and may be subject to response bias in hierarchical organizational cultures. High PRR scores may reflect social desirability or organizational pressure rather than the true PSC, particularly given the central argument of this study regarding potential underreporting and surface compliance.

The 5.3% adverse event reporting rate was derived from the administrative reporting records of one participating nursing home (a large government-operated facility, which also participated in the qualitative phase), rather than from staff self-reports. It was calculated as the proportion of frontline staff with at least one adverse event formally reported to facility management and documented in the facility’s reporting records during the 12 months preceding the survey: the numerator was the number of staff with at least one such documented report (*n* = 25), and the denominator was the total number of frontline staff at the facility during the same period (*N* = 476). The data were extracted retrospectively by the research team from the facility’s management department. Comparable reporting records could not be obtained from the other 10 nursing homes. Adverse events were documented according to the facility’s own reporting procedures rather than a standardized taxonomy, and events never brought to management’s attention could not be captured; underreporting therefore could not be estimated. The 5.3% rate should thus be interpreted as the percentage of staff who engaged in formal reporting behavior during the preceding year, not as the incidence of adverse events.

#### 2.3.2 Qualitative measures

For the qualitative phase, the researchers immersed themselves in the research setting to conduct focus group interviews. A self-developed semi-structured interview guide was formulated based on a literature review and the quantitative findings. The interview guide was developed in consultation with two senior nursing management specialists and a qualitative research expert, followed by pilot testing to ensure the comprehensiveness and depth of the content while allowing participants to freely express themselves. The core questions centered on the weaker PSC dimensions identified in the quantitative phase, aiming to explore underlying perceived reasons, staff perceptions, challenges faced, and suggestions for improvement (3).

The key questions in the interview guide included, but were not limited to: (1) “What are the most common safety hazard scenarios encountered in your work? What do you consider to be the most significant safety hazard? “(2) “Do you truthfully report safety hazards or adverse events when they are discovered or occur? What factors might hinder you from reporting?” (3) “Which work process designs do you feel pose operational risks or are unreasonable? What is your perspective on the actual effectiveness of the ‘non-punitive reporting’ system?” (4) “What measures do you believe could effectively reduce the underreporting and omission of adverse care events in nursing homes?” and (5) “What measures do you feel would genuinely improve patient safety and enhance patient safety culture?”

### 2.4 Data collection process

#### 2.4.1 Quantitative data collection

The questionnaire was administered to staff at 11 nursing homes in South China between August 2021 and January 2022. We adopted a hybrid approach combining electronic questionnaires (distributed via institutional WeChat work groups) and paper-based questionnaires (distributed by trained research assistants during morning briefings and deposited into sealed suggestion boxes after being completed by staff) to ensure accessibility and breadth of the data collection. All electronic questionnaires underwent independent review and cross-checking by two researchers to ensure data entry accuracy. For the paper questionnaires, data were entered into the computer by designated personnel and independently verified by a second researcher to ensure accuracy.

**Figure 1:**
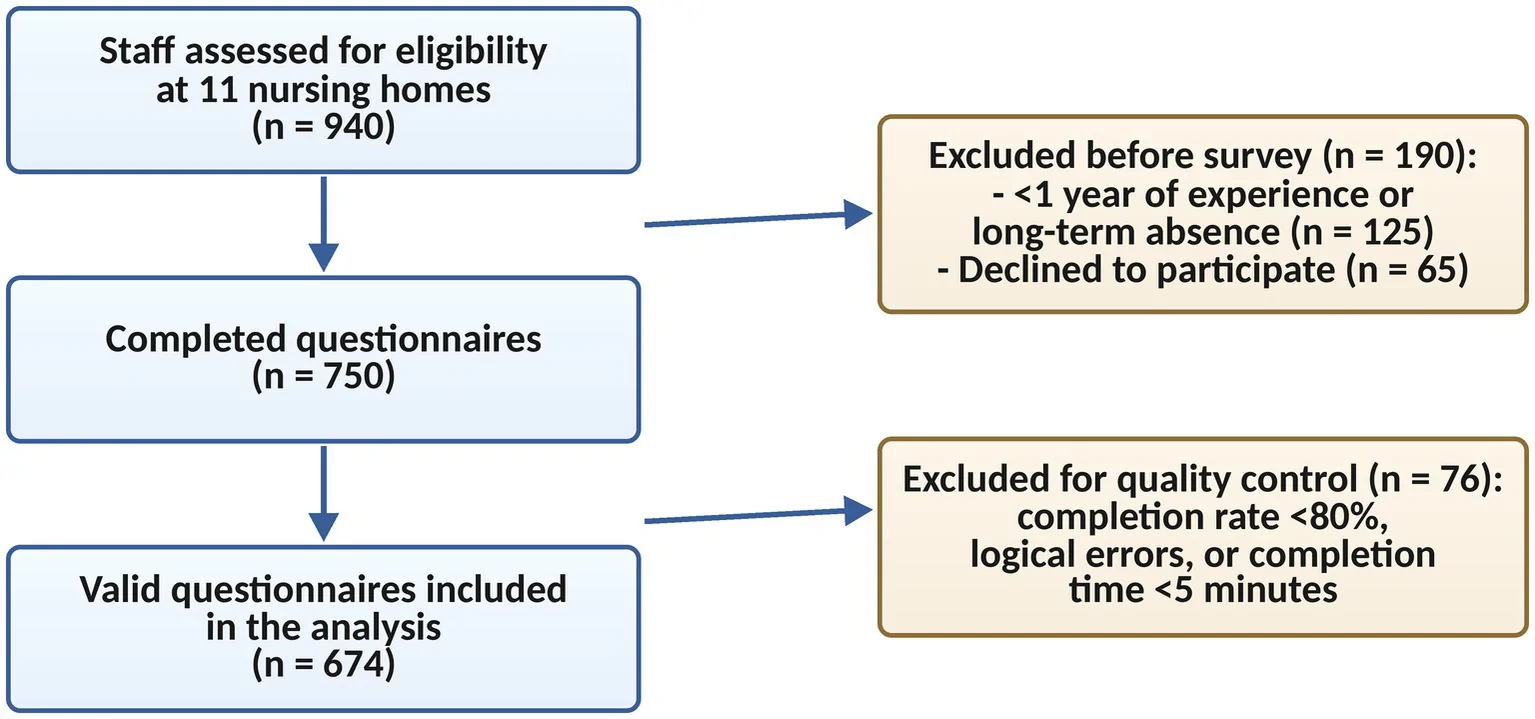
Flow diagram of quantitative participant recruitment.

Using a convenience sampling method, a cluster sampling approach was applied to survey 940 staff members across the 11 nursing homes. Based on the study criteria, we excluded 125 individuals (due to < 1 year of experience or long-term absence) and 65 individuals who declined to participate in the survey, resulting in 750 completed questionnaires. An additional 76 questionnaires were excluded due to completion rates below 80%, logical errors, or implausibly short completion times (< 5 min). From this parent sample, the present analysis included the subsample of 674 questionnaires with complete and valid NHSOPSC data (Figure 1); the derivation of the parent sample has been reported previously (10).

#### 2.4.2 Qualitative data collection

Qualitative data were collected through semi-structured focus group interviews (24). Interviews were facilitated by a male associate professor with extensive experience in qualitative research and assisted by an observer who took notes. The specific process was as follows:

1. Pre-observation (participant observation): The interviewer visited the nursing homes as a training instructor and collaborative researcher, participating in daily activities and interacting with interviewees to build rapport and mutual trust. This effort aimed to make observations flexible and non-intrusive (25), facilitating authentic expression from the participants. However, the interviewer’s dual role as a training instructor may have introduced social desirability bias and role-related influence. The participants may have viewed the interviewer as an expert or evaluator, especially in hierarchical institutions. To mitigate this, the interviewer (a) explicitly stated their independent researcher status and non-evaluative role at the beginning of each interview, (b) avoided discussing specific facility performance or management issues outside the interview context, (c) used self-deprecating and non-authoritative language to reduce status differential, and (d) encouraged participants to share negative experiences and assured them that criticism would not be reported to management. These steps were performed to enhance transparency regarding the interviewer’s positionality.
2. Development of a Focus Group Interview Guide: Interviews followed a semi-structured guide (see Section 2.3.2) with pre-set core questions but allowing participants free expression. Ethical considerations, including informed consent and confidentiality, were adhered to as described in Section 2.1.
3. Implementation and Quality Control of Focus Group Interviews: Interviews were conducted between May and June 2022 and were scheduled for approximately 60 min each (actual duration: 38–62 min, mean = 51 min). Seating was arranged in a hollow square layout, and refreshments were provided to create a relaxed and egalitarian atmosphere. Based on the research objectives, separate focus groups were conducted for nursing home managers, clinical frontline nurses, and caregivers to capture perspectives from different hierarchical levels and roles. The researcher initiated each session with a friendly greeting, explained the study’s purpose, and confirmed confidentiality of participation. Discussion topics were introduced according to the interview guide, with the sequence adjusted as appropriate based on the flow of conversation.

During the interviews, the researcher utilized effective communication skills, using language that was easily understood by the participants, minimizing interruptions, and asking probing or clarifying questions when necessary. The researcher maintained a neutral stance, supplemented by field notes that objectively recorded nonverbal cues.

Each interview was recorded by the moderator and the observer. At the conclusion of each interview, the researcher briefly summarized the main points and verified the recorded content with participants to ensure accuracy. Key themes were also verified with the participants at the end of each interview. Any unclear or ambiguous content was promptly clarified with the participants. Audio recordings were transcribed verbatim within 24 h. Full transcripts were not returned to participants after the session due to time constraints and participant availability. Finally, the participants were thanked for their support and given small gifts.

Sampling and interviews concluded when data saturation was reached (i.e., when interviews with two consecutive focus groups yielded no new themes or perspectives). The researcher documented their observations and personal feelings throughout the data collection and transcription process to enhance research reflexivity and inform the thematic analysis.

All interviews were conducted in Mandarin Chinese, the native language of the participants. Audio recordings were transcribed verbatim in Chinese by a trained research assistant. For inclusion in the manuscript, illustrative quotations were translated from Chinese to English by the first author (bilingual in Chinese and English) and independently backtranslated by the corresponding author to verify accuracy. Discrepancies in translation were resolved through discussion between the two translators. The translated quotations were checked against the original audio to ensure fidelity.

### 2.5 Data analysis

#### 2.5.1 Quantitative analysis

Quantitative data were analyzed using SPSS 25.0 software (IBM, United States). The quantitative phase was reported in accordance with the STROBE guidelines for cross-sectional studies. A completed checklist is provided in Supplementary Material 2. The PRR and scores for each dimension of the Chinese version of the NHSOPSC are reported using descriptive statistics, such as the mean and standard deviation (SD). Differences in total PSC scores across organizational characteristics (e.g., operational mode, ownership nature, and bed capacity) and participant characteristics (e.g., years of experience, income satisfaction, and job satisfaction) were analyzed using independent samples t-tests or one-way analysis of variance (ANOVA). For one-way comparisons, Levene’s test was used to assess homogeneity of variances. Welch’s ANOVA was utilized for *p*-values of < 0.05. Bonferroni adjustment was applied to post-hoc pairwise comparisons in the case of multiple comparisons.

Work experience in nursing home care was dichotomized as 1–3 versus ≥ 3 years in all descriptive, univariate, and regression analyses (Tables 1–3), consistent with the coding scheme used in the parent project (10). For the regression model, 1–3 years served as the reference category. For the regression analysis, the five-level income satisfaction and job satisfaction variables were collapsed into three categories (dissatisfied or very dissatisfied, neutral, satisfied or very satisfied) to ensure adequate cell sizes.

**Table 1:** Distribution of demographic and occupational characteristics of quantitative study participants (*n* = 674).

Characteristic category	Frequency (*n*)	Percentage (%)
Facility operation mode		
Government-operated	346	51.3
Privately-operated	328	48.7
Job position		
Caregivers	567	84.1
Registered nurses (RNs)	45	6.7
Technician/Rehabilitation Specialist	30	4.5
Physician	21	3.1
Social worker	11	1.6
Gender		
Female	622	92.3
Male	52	7.7
Age		
≥ 40 years old	462	68.5
< 40 years old	212	31.5
Work experience in nursing home care		
1–3 years	432	64.1
≥ 3 years	242	35.9
Hours worked per week		
> 40 h	663	98.4
≤ 40 h	11	1.6
Educational level		
Junior high school or below	317	47.0
High school/vocational school	313	46.4
Bachelor’s degree or above	44	6.5

**Table 2:** Univariate analysis of PSC score differences by organizational and personal characteristics (*n* = 674).

Characteristics	PSC Score (M ± SD)	t/F	*p*-value
Facility operation mode			
Government-operated	4.02 ± 0.41	0.90	0.37
Privately-operated	4.05 ± 0.45		
Facility size (beds)			
Big (> 500)	4.02 ± 0.41	1.02	0.36
Middle (201–500)	4.03 ± 0.43		
Small (≤ 200)	4.08 ± 0.47		
Work experience in nursing home care (years)			
1–3 years	4.06 ± 0.43	2.08	0.04^*^
≥ 3 years	3.99 ± 0.42		
Income satisfaction			
Very dissatisfied	3.77 ± 0.36	6.47	< 0.001^**^
Dissatisfied	3.98 ± 0.37		
Neutral	4.03 ± 0.43		
Satisfied	4.13 ± 0.40		
Very satisfied	4.06 ± 0.49		
Job satisfaction			
Very dissatisfied	3.77 ± 0.39	5.90	< 0.001^**^
Dissatisfied	3.83 ± 0.33		
Neutral	4.00 ± 0.42		
Satisfied	4.10 ± 0.43		
Very satisfied	4.14 ± 0.44		

^*^p < 0.05; ^**^p < 0.01.

**Table 3:** Multiple linear regression analysis of factors associated with patient safety culture in nursing homes (*n* = 674).

Predictor	B (unstandardized)	SE	*β* (standardized)	*t*	*p*	95% CI
Constant	3.872	0.069	−	55.794	< 0.01**	[3.736, 4.008]
Income satisfaction						
Dissatisfied or very dissatisfied	−	−	−	−	−	−
Neutral	0.132	0.062	0.155	2.134	0.033*	[0.010, 0.254]
Satisfied or very satisfied	0.174	0.075	0.189	2.331	0.020*	[0.028, 0.320]
Job satisfaction						
Dissatisfied or very dissatisfied	−	−	−	−	−	−
Neutral	0.104	0.081	0.124	1.282	0.200	[−0.055, 0.263]
Satisfied or very satisfied	0.176	0.088	0.208	1.995	0.046*	[0.002, 0.350]
Years of work experience in nursing home care						
1–3 years	−	−	−	−	−	−
≥ 3 years	−0.098	0.040	−0.113	−2.456	0.014^*^	[−0.178, −0.018]

^a^F = 71.901, *R*^2^ = 0.350, adjusted *R*^2^ = 0.345; VIF max = 1.48 (< 5), Shapiro–Wilk *p* = 0.21 (residuals normal), Breusch–Pagan *p* = 0.18 (homoscedastic). ^b*^p < 0.05; ^**^p < 0.01. ^c^The reference group for income satisfaction was dissatisfied or very dissatisfied with income, the reference group for job satisfaction was dissatisfied or very dissatisfied with the job, and the reference group for years of work experience in nursing home care was 1–3 years. d. B = unstandardized regression coefficient; SE = standard error; β = standardized regression coefficient; CI = confidence interval; VIF = variance inflation factor. Dashes (−) indicate statistics that are not estimated for reference categories.

No automated stepwise selection procedure was used. The independent variables were pre-specified based on prior PSC theory and literature (7, 10, 18, 26). The candidate predictors included facility operation mode, facility size, years of work experience, income satisfaction, and job satisfaction. Variables that showed an association with the total PSC score in the univariate analysis (*p* < 0.05) were entered simultaneously into a multiple linear regression model. The dependent variable was the total PSC score. Categorical variables were dummy-coded, with reference categories specified as follows: dissatisfied or very dissatisfied was the reference group for income satisfaction, dissatisfied or very dissatisfied was the reference group for job satisfaction, and 1–3 years was the reference group for years of work experience in nursing home care.

Normality and collinearity tests were performed to ensure that model assumptions were met. The variance inflation factor (VIF) was used to evaluate multicollinearity, with a VIF of > 5 indicating moderate collinearity and a VIF of > 10 indicating severe collinearity (27). All independent variables had VIF values below 2.5 (maximum VIF = 1.48), indicating no significant collinearity. Both unstandardized regression coefficients (B) and standardized regression coefficients (β) were reported for each predictor, along with 95% confidence intervals (CI), t-values, and *p*-values. The model diagnostics include the F-statistic, R^2^, adjusted R^2^, and residual analyses (Shapiro–Wilk test for normality of residuals, Breusch–Pagan test for homoscedasticity).

The 674 participants were nested within 11 nursing homes (range: 38–92 staff per facility, mean = 61.3, SD = 15.4). We acknowledge that staff responses may be correlated due to shared organizational leadership, staffing patterns, institutional culture, ownership, policies, and reporting systems. The regression analysis treated all staff members as independent observations, which is a study limitation. We did not use multilevel modeling, generalized estimating equations, or cluster-robust standard errors because the number of clusters (n = 11) was insufficient for reliable multilevel estimation and the intraclass correlation coefficient was not calculated. We conducted a sensitivity analysis by running the regression model with and without facility-level dummy variables. The pattern of significant predictors remained consistent, and the coefficients changed by less than 10%. We report this as a robustness check but acknowledge that standard errors may be underestimated and *p*-values may be overly optimistic due to unaccounted clustering. Future research with a larger number of facilities should employ multilevel modeling to account for nesting.

All statistical tests were two-tailed, with a *p*-value of < 0.05 considered statistically significant. Given the cross-sectional design of the study, the regression analysis identified statistical associations, not causal relationships.

#### 2.5.2 Qualitative analysis

Thematic analysis was used to code and extract themes from the qualitative interview data, assisted by NVivo 14 software in this secondary analysis to ensure systematicity and traceability. Coding was performed by two independently trained researchers and strictly followed the six-phase process of the thematic analysis framework outlined by Braun and Clarke (28). The analysis process was iterative and involved the following steps: (1) familiarization with the data through repeated reading of transcripts; (2) generating initial codes line by line to capture core experiences, perceptions, and latent meanings; (3) searching for themes by comparing, inducing, and categorizing codes into potential themes and subthemes; (4) reviewing themes to assess internal homogeneity and external heterogeneity; (5) defining and naming themes to clarify core content and boundaries with concise, accurate labels; and (6) producing a report with representative extracts. Regular peer debriefing sessions were held for the three researchers to discuss and refine the coding framework and emerging themes and enhance the trustworthiness of the analysis. Any discrepancies during coding were resolved through consensus meetings, which included a third expert if necessary to ensure objectivity. Inter-coder reliability was verified using Cohen’s kappa coefficient. The obtained kappa value was 0.84, indicating almost perfect agreement (29).

Our software-aided process, which strictly adhered to the thematic analysis framework and combined dual coding with a consensus-based resolution of discrepancies, ensured the depth and credibility of our analysis and met quality standards for qualitative research (28–30).

The qualitative phase was reported in accordance with the Consolidated Criteria for Reporting Qualitative Research (COREQ) 32-item checklist (30). The key items addressed were research team characteristics (interviewer training and experience), study design (theoretical framework, participant selection, and setting), data collection (interview guide development, field notes, data saturation, and transcript return), and analysis (number of coders, coding description, theme derivation, participant checking, and inter-coder reliability). A completed COREQ checklist is provided in Supplementary Material 2.

#### 2.5.3 Mixed-methods integration

A mixed-methods design aims to integrate quantitative and qualitative data. In the quantitative phase of this study, we identified weak dimensions of PSC and factors associated with them using a questionnaire. The qualitative data were then re-examined with a focus on these weak dimensions.

We integrated the quantitative and qualitative data using “connecting,” “explaining,” and “expanding” strategies, which are detailed below.

Connecting: The connection between the two datasets occurred at the analysis and interpretation stage: the secondary thematic analysis of the interview corpus was oriented by the weak PSC dimensions identified in the quantitative results. For example, the dimension Staffing was targeted for qualitative inquiry due to its low PRR score (67.0%). For this dimension, qualitative themes such as the “inability to complete nursing tasks according to standards due to excessive workload” and “challenges in staff adaptation due to high turnover rates” (not less than 40% within 1 year) were analyzed and discussed in conjunction with the quantitative data.

Explaining: Qualitative data were utilized to explain the statistical relationships revealed in the quantitative research. For example, given that the Staffing dimension had the lowest PRR of the six dimensions (67.0%), qualitative interviews examined how staff shortages were associated with work pressure, willingness to report, and safety behaviors. The interviews uncovered specific barriers (e.g., manpower gaps during acute event handling and inter-professional role conflicts). These qualitative findings provide a contextual explanation for the quantitative associations, detailing possible explanatory pathways for weak PSC dimensions and cross-validating the results.

Expanding: We constructed a comprehensive improvement framework to further integrate the quantitative and qualitative findings. Qualitative data extended the quantitative findings by providing richer, more nuanced background information and contextual understanding, facilitating the construction of a more holistic PSC improvement framework. For instance, for the Staffing issue, the findings propose a comprehensive approach that includes optimizing caregiver ratios, strengthening interdepartmental collaboration, and enhancing staff coping capabilities through simulation training. Through such integration, this study aimed to transcend the limitations of mono-methodological approaches to provide a more comprehensive understanding of PSC in nursing homes and an informed foundation for developing effective interventions.

A table of quantitative weak dimensions, specific items, qualitative themes, illustrative quotations, interpretation, and practical implications is provided in Supplementary Material 1. This joint display directly demonstrates how qualitative data explain the quantitative results, particularly for the weak dimensions of Staffing and Adherence to Workflow, as well as the low adverse event reporting paradox.

## 3 Results

This study employed a mixed-methods approach to explore the status, associated factors, and possible explanatory pathways of PSC in 11 nursing homes across South China.

### 3.1 Participant characteristics

#### 3.1.1 Quantitative analysis participants

The quantitative phase included 674 frontline staff members from 11 nursing homes. As shown in Table 1, the participants predominantly comprised caregivers (84.1%), followed by licensed professional staff (15.9%), which included physicians (3.1%), RNs (6.7%), technicians/rehabilitation specialists (4.5%), and social workers (1.6%). The sample was majority female (92.3%) and generally older, with 68.5% of participants aged ≥ 40 years. The work hours were extensive, with 98.4% of participants reporting working > 40 h per week. In terms of tenure, 35.9% (n = 242) of the staff had been engaged in nursing home care for ≥ 3 years. Educational attainment among the participants was generally low: 317 individuals (47.0%) held a junior high school education level or below, 313 individuals (46.4%) had a high school or technical secondary school education level, and only 44 individuals (6.5%) possessed a bachelor’s degree or higher. Notably, caregivers constituted the majority of the sample (84.1%), whereas RNs accounted for only 6.7%. Therefore, the quantitative findings primarily reflect the perspectives of caregivers rather than a balanced multidisciplinary staff sample.

#### 3.1.2 Qualitative analysis participants

A total of 35 staff members participated in six focus group interviews (Supplementary Table 1). The participants were recruited from one government-operated and one privately-operated nursing home, comprising 12 caregivers, 12 RNs, and 11 managers. The sample included 4 males and 31 females, with ages ranging from 23 to 61 years. The interview duration varied from 38 to 62 min, with an average length of 51 min. Work environment, years of experience in nursing home care, and job positions varied among the participants.

Their demographic characteristics (age, gender, and educational background) spanned a broad range, and maximum variation sampling deliberately covered roles underrepresented in the quantitative sample (RNs and managers), supporting the diversity and complementarity of the qualitative sample.

### 3.2 Current status of patient safety culture in nursing homes

#### 3.2.1 PRR and dimension scores

The overall PSC PRR was 86.5% (Table 4). Ranked from high to low, the PRR for each dimension was: Organizational Learning and Continuous Improvement (97.6%), Handoff and Communication (94.9%), Management Support for Safety (93.5%), Teamwork (93.1%), Adherence to Workflow (72.7%), and Staffing (67.0%). As they had a PRR of > 75%, the first four dimensions were classified as areas of strength, while Adherence to Workflow and Staffing fell into the intermediate category. No dimensions were classified as areas for improvement (i.e., PRR < 50%) (31).

**Table 4:** PRR and scores of various dimensions of patient safety culture in nursing homes (*n* = 674).

Patient safety culture	PRR (%)	Mean±SD
Overall	86.5	4.08 ± 0.42
Dimensions		
Organizational learning and continuous improvement	97.6^*^	4.29 ± 0.50
Handoff and communication	94.9^*^	4.20 ± 0.46
Management support for safety	93.5^*^	4.18 ± 0.52
Teamwork	93.1^*^	4.17 ± 0.56
Adherence to workflow	72.7	3.74 ± 1.10
Staffing	67.0	3.64 ± 0.62

PRR, positive response rate; *PRR > 75%, classified as areas of strength.

#### 3.2.2 PRR and item scores

Among the 23 NHSOPSC questionnaire items, 18 had a PRR of > 75%. Four items fell within the intermediate range (50% ≤ PRR ≤ 75%), while one item (“Due to the high turnover of staff in this institution (not less than 40% within 1 year), it is difficult to ensure the safety of the older residents”) was classified as needing improvement (PRR = 48.5%, mean score = 2.89 ± 1.21).

#### 3.2.3 Univariate analysis of factors associated with patient safety culture in nursing homes

Next, we compared the associations between different organizational characteristics and participants’ personal characteristics and the total PSC score (Table 2). PSC scores did not differ significantly between groups based on organizational characteristics such as the operation mode (*p* = 0.37) or the bed number of the nursing home (*p* = 0.36), indicating that these macro-organizational factors were not significantly associated with the overall PSC. However, several personal characteristics were significantly associated with PSC, including years of work experience (*p* = 0.04), income satisfaction, and job satisfaction (both *p* < 0.001).

#### 3.2.4 Multiple linear regression analysis of factors associated with patient safety culture in nursing homes

The multiple linear regression revealed that income satisfaction, job satisfaction, and years of work experience in nursing home care were statistically associated with PSC (Table 3).

For income satisfaction, a dose–response pattern was observed relative to the dissatisfied or very dissatisfied reference group: staff who were neutral [B = 0.132, β = 0.155, 95% confidence interval (CI) (0.010, 0.254), *p* = 0.033] and those who were satisfied or very satisfied [B = 0.174, *β* = 0.189, 95% CI (0.028, 0.320), *p* = 0.020] reported progressively higher PSC scores. Similarly, staff who were satisfied or very satisfied with their job had higher PSC scores than the dissatisfied reference group [B = 0.176, *β* = 0.208, 95% CI (0.002, 0.350), *p* = 0.046], whereas the neutral group did not differ significantly [B = 0.104, *β* = 0.124, 95% CI (−0.055, 0.263), *p* = 0.200]. Longer work experience in nursing home care was associated with lower PSC scores [B = −0.098, *β* = −0.113, 95% CI (−0.178, −0.018), *p* = 0.014, reference group: 1–3 years]. This model explained 35.0% of the variance in total PSC scores (*R*^2^ = 0.350, adjusted *R*^2^ = 0.345), indicating that individual-level satisfaction and relevant work experience were statistically associated with PSC in nursing homes. Model diagnostics indicated no substantial multicollinearity (maximum VIF = 1.48), normally distributed residuals (Shapiro–Wilk *p* = 0.21), and homoscedasticity (Breusch-Pagan *p* = 0.18).

### 3.3 Qualitative results

Following the six-step thematic analysis method proposed by Braun and Clarke (28), we coded and extracted four core themes and 12 secondary sub-themes. The qualitative findings are presented in a theme-subtheme-evidence chain format. All interview materials were anonymized. The citation numbering for participant interviews was as follows: FG was used to denote the focus group. Participants’ roles were abbreviated as Manager (M), Nurse (N), and Caregiver (C). The first digit represents the sequence number of the focus group, while the second digit indicates the anonymous number of the respondent within that focus group. For instance, C FG2-02 refers to the second caregiver in the second focus group.

#### 3.3.1 Core themes

##### 3.3.1.1 Theme 1: safety anxiety associated with workforce shortages

This theme corresponds to the PRR of 67.0% in the Staffing dimension of quantitative research, reflecting the insufficient allocation of human resources in the surveyed nursing homes and the associated patient safety concerns. The sub-themes are as follows:

1. Contradiction between workload and adherence to standards: Multiple caregivers (frontline staff primarily responsible for assisting older residents with daily living) described that excessive workloads hindered their ability to strictly follow protocols. For instance, one stated, “During the day shift, there is only 1 RN and 3 caregivers to care for 45 disabled older individuals, and it is truly impossible to turn them every 2 hours.” (C FG1-02)
2. Nighttime caregiver shortages and emergency dilemmas: Insufficient staffing during night shifts in treatment areas poses high risks, increasing difficulty responding to emergencies and causing psychological stress among employees. One participant described: “At 3 AM, an older resident fell while using the restroom. Another caregiver went to call the on-duty doctor, and at the same time, multiple residents were calling for assistance. I was running between several residents, and in that moment, I just wanted to cry.” (C FG2-05)

##### 3.3.1.2 Theme 2: non-standardized workflows and interprofessional role conflicts

This theme is correlated with the quantitative finding of a PRR of 72.7% in the Adherence to Workflow dimension. It reveals the negative association of unclear workflows and ambiguous responsibilities with the culture of patient safety. The sub-themes are as follows:

1. Disconnection between written procedures and practice: Interviews revealed that written standard operating procedures (SOPs) lack effective training and demonstration, and employees described simplifying these processes, a pattern associated with weaker adherence to workflow. For example: “The SOP outlines 17 steps for oral care, but no one has demonstrated it. We relied on our own exploration and ultimately simplified it to three steps.” (C FG1-03)
2. Ambiguous and shifting responsibilities: Employees lack clarity regarding their respective responsibilities, leading to blurred professional boundaries associated with disrupted team collaboration and continuity of patient care. For instance: “The physician’s orders are to ‘assess the skin and turn the patient every 2 hours’, but the night shift nurse believes that is the aide’s responsibility, while the aide states ‘I cannot assess the grading of pressure injuries (N FG3-02)’.
3. Challenges associated with high employee turnover and insufficient training: High employee turnover is associated with challenges to workflow execution and patient safety, which is particularly manifested by the phenomenon of new employees without adequate training. For instance: “3–4 people leave every month, and new hires are trained for only 3 days before they directly take over shifts, yet many remain unfamiliar with workflows.” (C FG1-01) “Some employers intentionally do not retain veteran employees because new hires have a probation period and are cheaper!” (M FG5-01)
4. Challenges associated with family expectations: Requests from residents’ families often increase the complexity of providing care, sometimes even contradicting professional medical advice, a source of distress for frontline staff. For instance: “The family insists that ‘water must be given every 30 minutes,’ while we administer it according to the medical advice of Q2H, resulting in the family complaining that ‘you are inhumane (C FG2-04)’.

##### 3.3.1.3 Theme 3: cultural barriers to adverse event reporting

This theme corresponds to the paradox between the high overall PSC PRR (86.5%) and the low formally documented adverse event reporting rate (5.3%) obtained from facility records. It reveals an association between organizational silence and adverse event reporting within the context of Chinese culture and various reporting barriers, such as punishment, concerns about reputation, and external pressures:

1. The lack of an effective feedback mechanism is associated with a reduced willingness to report. After adverse event reporting, if the management does not provide proactive feedback and fails to implement improvement measures, employees may perceive reporting as meaningless, which may be associated with reduced willingness to make further reports. For instance: “Last time when an older resident fell, the nursing department responded with ‘Why were not you more careful?’ providing only criticism without any guidance or assistance. Since then, everyone believes that ‘reporting is useless,’ and no one in the department is willing to report anymore.” (N FG3-03)
2. Face culture and concerns about professional reputation: Face culture within the context of Chinese culture, along with anxiety surrounding negative evaluations, is associated with a reduced willingness of staff to admit mistakes, thus limiting opportunities to learn from errors. For instance: “Admitting to giving the wrong medication in the morning meeting? People will think your skills are poor, and your chances for promotion will definitely be jeopardized.” (N FG3-02) At the same time, respondents expressed concerns about their career prospects: “If it is due to my violations, I would definitely worry about the impact on my future.” (N FG4-05)
3. Punitive management and interpersonal relationship stress: Employees expressed general concerns about being criticized, having bonuses deducted, facing legal accountability, and damage to colleague relationships. These potential negative consequences are associated with reduced reporting behavior. For instance: “For any reason, every time an older resident falls, the on-duty staff is fined 100 RMB ($13.9), which affects the overall group’s performance.” (N FG3-02) “Everyone is afraid of being criticized, having bonuses deducted, and getting involved in lawsuits.” (M FG5-02) “Family disgrace should not be aired; I do not want other departments to know.” (N FG4-02) “Fearing it will affect relationships, if my colleagues do not report, I will not report either.” (N FG3-04)
4. Negative association of external pressures with reporting behavior: Concerns regarding family complaints and potential requests for compensation are associated with staff being more inclined to handle issues privately than report them through formal channels. “The first thing the family said was, ‘You must compensate us.’ Therefore, for events that are not obvious, there is a general tendency to handle them privately and not report them to the system.” (C FG1-03)

##### 3.3.1.4 Theme 4: staff-driven strategies for improving patient safety

This theme consolidates the improvement suggestions proposed by respondents based on their frontline experience, providing empirical findings for developing targeted interventions:

1. Establishing non-punitive reflection and learning spaces: Employees suggest creating a safe, non-punitive environment that encourages sharing experiences and learning, facilitating the extraction of lessons from mistakes. “We can learn from the adverse events reported by others (a lesson learned from a setback).” (M FG5-05) “It is recommended to hold a ‘near-miss’ sharing session once a month, without assigning blame, focusing solely on how to mitigate risks.” (M FG6-01)
2. Implementation of interprofessional scenario simulation training: The proposed training would promote collaboration among different roles and enhance the team’s ability to respond to emergencies, and foster communication and understanding across departments. For instance: “By having doctors, RNs, and caregivers simulate emergency situations during night shifts, they come to understand each other’s challenges.” (N FG4-01)

#### 3.3.2 Summary of qualitative research findings

Overall, the qualitative data provided a nuanced understanding of employees’ perceptions and experiences regarding PSC in nursing homes in South China. These insights highlight that, although positive aspects of PSC exist, underlying structural weaknesses are prevalent and primarily associated with limited human resources and non-standardized workflows. The perennial lack of personnel was linked to frontline staff resorting to *ad hoc* solutions to complete their daily tasks. Concurrently, ambiguous processes and interprofessional role conflicts were associated with a widening discrepancy between adherence to workflow requirements and real-world operations. Culturally embedded factors such as face culture, significant power distances, punitive management approaches, and concerns about family member complaints are further associated with reduced reporting of adverse events, contributing to widespread organizational silence. Employees proactively suggested locally relevant improvement strategies, including the establishment of non-punitive reflective spaces and the implementation of interprofessional scenario simulations to address these challenges.

## 4 Discussion

This study employed a mixed-methods approach to systematically analyze the status, factors associated with and possible explanatory pathways underlying PSC in nursing homes in South China. First, we quantitatively characterized the overall PSC and its dimensional performance. Additional qualitative findings showed the complex organizational behavior logic and unique sociocultural roots contributing to the identified PSC weaknesses. This integrative approach provides empirical evidence for enhancing patient safety management in nursing homes while also offering contextual insight to deepen the understanding of PSC theory, particularly in the context of non-Western cultures (20).

### 4.1 Overall level and dimensional characteristics of PSC in nursing homes

This study found an overall PRR of 86.5% and a mean PSC score of 4.08 ± 0.42 for nursing homes in South China. These results align with the evaluations of PSC in nursing institutions both domestically and internationally (3, 31), indicating that most nursing home staff hold a positive attitude toward patient safety. Notably, the overall PSC score in this study is slightly higher than that reported in a prior study of public hospitals in China (32). This may be attributed to intrinsic differences in service models between nursing homes, which emphasize daily care and interpersonal communication, and comprehensive hospitals, which feature high-pressure, technology-intensive environments. Such a model may facilitate the high PRR (all > 75%) in dimensions such as Handoff and Communication, Management Support for Safety, and Teamwork, with a particularly high PRR of 97.6% for Organizational Learning and Continuous Improvement. These results are consistent with the core role of organizational learning in the construction of PSC (33), and PRR scores may have benefited from the relatively flat management structure and daily experience exchange mechanisms in nursing homes. However, the high PRR values may partly reflect surface compliance or social desirability rather than a uniformly strong PSC. Therefore, PRR thresholds were used as heuristic benchmarks rather than definitive classifications of culture quality (see Section 4.3).

In contrast, the PSC dimensions Adherence to Workflow and Staffing were relatively weak, with PRRs of 72.7 and 67.0%, respectively, placing them in the intermediate range. This finding aligns with multiple studies highlighting the common phenomenon of insufficient human resources and challenges in process execution within nursing homes (31, 34). Research indicates that disparities among different PSC dimensions may be constrained by the complexity of workflows and difficulties in staff recruitment, which are particularly pronounced in nursing homes (35). Compared to developed Western countries, nursing homes in China exhibit significant gaps in the specialization of care staff, training systems, and compensation and benefits, which may contribute to the Staffing dimension’s emergence as a prominent weak link (6, 36). At the same time, the lack of mature industry standards and regulatory systems in China presents unique challenges to workflow adherence.

Notably, 98.4% of the participants reported working more than 40 h per week. This near-universal pattern of extended working hours is consistent with the qualitative accounts of excessive workload, night-shift strain, and safety anxiety described under Theme 1, and suggests that chronic understaffing may have become normalized in these settings. Sustained long working hours are associated with fatigue and occupational burnout, which may further weaken adherence to safety practices. However, the lack of variation in this variable in the present sample prevented a more granular analysis of workload in relation to PSC (see Section 4.6).

### 4.2 Core factors associated with PSC and underlying perceived pathways

The multiple linear regression analysis indicated that staff income satisfaction, job satisfaction, and years of work experience in nursing home care are factors statistically associated with PSC in nursing homes. Higher levels of income satisfaction and job satisfaction were associated with higher total PSC scores, which aligns with several studies suggesting a positive correlation between employee satisfaction and PSC (26). Employees with high satisfaction typically exhibit greater work engagement, a stronger sense of organizational identity, and more proactive safety behaviors, all of which may be associated with enhanced PSC.

However, we found that a longer duration of work in nursing home care was associated with a lower total PSC score, which contradicts some research findings (37). This paradox may indicate that employees working in nursing home environments for extended periods are prone to occupational burnout and psychological exhaustion through continuous exposure to resource scarcity and imperfect work processes (38). Specifically, sustained exposure to high-pressure, resource-scarce environments is associated with occupational burnout, which may gradually erode employees’ innovative drive and confidence in organizational improvements, ultimately fostering a negative perception of PSC (39). As employees’ experience increases, they may feel disillusioned by attempts at organizational improvements, develop a “taken for granted” mindset regarding existing issues, or experience enhanced resistance to change due to organizational inertia, leading to more negative perceptions of PSC. This phenomenon may be particularly pronounced in the context of China’s rapidly developing care service industry for older adults, where supporting mechanisms are not fully established. Senior employees may have experienced multiple stages of industry development, leading to a deeper understanding of existing issues. However, these individuals often lack effective feedback channels or motivation to improve. This can result in learned helplessness, a psychological state in which individuals come to believe they are unable to control or change their circumstances, even when opportunities for change exist. For instance, if senior staff repeatedly attempt to report safety concerns or suggest improvements but receive no positive feedback or see no tangible changes, they may develop this sense of futility and stop trying, rendering them more pessimistic about PSC. In a cultural context that emphasizes collectivism and authority compliance, senior employees may be more inclined to accept the status quo within the organization rather than actively attempt to implement improvements. For instance, in Chinese nursing homes, high-power distance may suppress employees’ willingness to provide feedback, with long-standing organizational inertia further exacerbating resistance to change. To prevent the development of negative perceptions of PSC among experienced staff, organizations such as nursing homes may need to adopt measures to enhance professional recognition and psychological support for senior employees.

Our qualitative findings provide a perceived explanatory pathway for the low dimensional scores observed in the quantitative results. For example, Theme 1, Safety Anxiety Associated with Workforce Shortages, directly corroborates the weaknesses in the Staffing dimension. Respondents commonly reported excessive workloads, leading to profound concerns about patient safety. This ongoing shortage of manpower was linked to high staff turnover, with new employees entering work without sufficient familiarity with workflows, a pattern associated with heightened safety risks (32). Similarly, Theme 2, Non-Standardized Workflows and Interprofessional Role Conflicts, elucidates the reasons underlying the low Adherence to Workflow dimension score. Despite written SOPs, employees described that they had not been sufficiently demonstrated, indicating a lack of enforceability of SOPs. Additionally, a lack of clarity regarding the responsibilities of doctors, RNs, and caregivers was associated with responsibility shifting, while requests from residents’ families often complicated workflows, making it challenging for frontline staff to strictly adhere to established processes and contributing to informal workarounds (“creative noncompliance”) that were linked to safety hazards. These findings deepen the applicability of PSC theory in non-Western cultural contexts and offer contextually grounded explanations for how organizational management, human resources, and cultural factors relate to weaknesses in PSC in nursing home settings.

As noted in Sections 2.2.1 and 3.1.1, the regression findings and qualitative interpretations discussed above primarily reflect caregiver perspectives on PSC rather than balanced multidisciplinary staff perspectives. Role differences in PSC, reporting behavior, and workflow adherence are likely important, and future research should consider stratifying results by professional role or examining role-specific patterns.

### 4.3 Patient safety culture and organizational silence: underlying barriers to adverse event reporting

Although the quantitative results of this study indicate a high overall PRR for PSC (86.5%), the formally documented adverse event reporting rate was very low: facility reporting records from one government-operated nursing home showed that only 5.3% of its frontline staff had at least one adverse event formally reported to management in the 12 months preceding the survey (see Section 2.3.1 for the full definition and data source). This apparent contradiction can be better understood through Theme 3, Cultural Barriers to Adverse Event Reporting.

The common perception that “reporting is futile” is a significant contributor to organizational silence. Respondents indicated that when adverse events are reported, there is a lack of positive feedback and substantive improvement measures from management, and reporting may instead be met with blame. These findings closely relate to the absence of a non-punitive reporting culture (38). When employees perceive that reporting behavior will not be followed by positive changes and may even be associated with negative consequences, their willingness to report may decrease. Thus, prioritizing improvements in feedback mechanisms and communication regarding event-related improvements is crucial to enhance voluntary employee reporting of patient safety incidents. Previous research (40) suggests that focusing on these aspects may be more effective than other forms of cultural change.

Reluctance to admit mistakes among employees was associated with face culture and high-power distance, both of which were linked to reduced reporting of adverse events (9). These cultural dynamics illustrate how core elements of PSC may be shaped by traditional Confucian concepts, such as the “doctrine of the mean” and “harmony is precious.” In high-power distance environments that emphasize collective harmony and conflict avoidance, employees may tend to maintain superficial harmony and avoid reporting errors or challenging authority, fearing that being perceived as “the nail that sticks out” could harm interpersonal relationships or career advancement (7). Within Confucian culture, personal reputation and social evaluation are crucial; employees may conceal errors out of concern that admitting mistakes could damage their image or career prospects (41). These dynamics highlight the importance of developing transparent communication channels and fostering an inclusive PSC (15).

The paradox of high PSC scores coupled with low reporting rates is accounted for by the concept of “surface compliance and deep silence,” in which staff outwardly endorse safety culture while inwardly suppressing adverse event disclosure. This phenomenon suggests that employees may report a positive perception of PSC in surveys to align with organizational expectations or social norms (i.e., surface compliance) yet actually avoid formally reporting adverse events due to deeply ingrained cultural and organizational barriers. In contexts characterized by high-power distance and face culture, frontline employees may be inclined to report PSC perceptions aligned with organizational expectations or social norms in surveys to avoid potential negative evaluations or conflicts (41). However, when confronted with the actual reporting of adverse events, they may conceal or not report issues for various reasons, such as fear of punishment, damage to professional reputation, interpersonal relationship pressures, and complaints from family members, ultimately resulting in silence. This phenomenon should be distinguished from related but distinct concepts: organizational silence refers to the collective withholding of information by employees, psychological safety concerns the perceived safety of interpersonal risk-taking, impression management involves strategically controlling self-presentation, and social desirability bias is a response tendency in self-report measures. The phenomenon of surface compliance and deep silence specifically captures the disconnect between positive survey responses and actual reporting behaviors in high-power distance, face-culture contexts, as evidenced by the coexistence of an 86.5% PRR and a 5.3% reporting rate in this study.

Reluctance to report falls due to fear of family complaints reveals how external pressures, particularly the risk of family complaints and economic consequences, are associated with adverse event reporting behavior. Lee et al. examined the association between family complaints, economic compensation risks, and reporting behavior (8), finding that external pressure was linked to stronger intrinsic cultural resistance. In contexts without comprehensive medical dispute resolution mechanisms or a foundation of trust, frontline staff may opt to handle adverse events privately to avoid trouble and liability, rather than reporting them through formal channels. This external pressure interacts with internal cultural factors, collectively contributing to the perceived explanatory pathways that underlie the low rates of adverse event reporting in Chinese nursing homes. Specifically, the lack of a supportive organizational culture, concerns among employees, and external pressures contribute to a pervasive phenomenon of organizational silence.

### 4.4 Employee-driven improvement pathways and systematic intervention strategies

Although the qualitative findings of this study highlighted many challenges, they also showed that frontline staff are intrinsically motivated and have suggestions for enhancing PSC. These practice-based recommendations can provide empirical findings to inform the design of more systematic and hierarchical intervention strategies.

The suggestion to establish a non-punitive reflection and learning space directly addresses the core need to improve workplace psychological safety. A safe, open communication environment would encourage employees to share near-miss experiences rather than attribute blame. A non-punitive reflective space may help mitigate power asymmetries, encourage voluntary reporting by employees, and reduce organizational silence, thereby supporting both adverse event reporting and organizational learning.

The recommendation to implement interprofessional simulation training highlights the critical role of teamwork and situational awareness training in enhancing patient safety. By simulating high-risk scenarios, such as emergency events during night shifts, employees with different roles can better understand each other’s responsibilities, challenges, and needs, thereby fostering interprofessional communication and collaboration and enhancing the team’s ability to respond to emergencies. As demonstrated by previous studies (23), simulation training is an effective strategy for practicing teamwork, collaboration, and communication skills during nursing handoffs.

Based on the integration of the findings of this study, we suggest the following strategies as hypotheses or priorities for future intervention development and evaluation in nursing homes. They were suggested by participants and remain to be tested:

Cultural Dimension: One potential priority is to actively cultivate a non-punitive safety reporting culture. Managers could establish open dialog and transparent feedback mechanisms to reduce organizational silence associated with employees’ fears of losing face or believing that reporting is futile. Simultaneously, leveraging the positive aspects of the Eastern culture of harmony and unity over conflict, teams could promote a culture of speaking up, reporting, learning, patient-centeredness, and justice to collaboratively solve problems rather than resorting to blame.

Institutional Level: First, optimizing the allocation of human resources could help create appropriate ratios of caregivers to residents. One possibility is to introduce smart caregiving devices to alleviate pressure associated with staff shortages. Second, a comprehensive employee career development and psychological support system could be established to mitigate occupational burnout among senior staff, such as multi-level promotion pathways for caregivers and ongoing education and psychological counseling services. Third, drawing on previous experiences (42), standardized workflows could be implemented that emphasize leadership support and full participation alongside multifaceted patient safety plans and ongoing education, such as regular scenario simulation training.

Leadership Dimension: Another potential priority is to strengthen the safety leadership of nursing home managers. Managers could be encouraged to directly engage with frontline staff, establish trust, and promptly address their safety concerns and suggestions while providing necessary support. This approach might enhance employees’ organizational identification and job satisfaction. In cultural contexts characterized by high-power distances, leaders could proactively aim to bridge power gaps, promote two-way communication, and transform punitive mechanisms into learning opportunities. The proposals from frontline staff reflect localized practical wisdom and offer empirically grounded directions for the design of future PSC interventions in nursing homes.

### 4.5 Explanatory power of multiple regression models and their extensions

The multiple regression analysis in this study revealed that income satisfaction and job satisfaction were positively associated with total PSC scores, whereas more years of work experience in nursing home care were negatively associated with PSC scores. Overall, the model explained 35.0% of the variance in the total PSC score (*R*^2^ = 0.350, adjusted *R*^2^ = 0.345). Although this explanatory power is comparable with similar studies, there are several possible sources of the unexplained variation. First, employees’ perceptions of PSC are significantly associated with micro-individual experiences (such as income and job satisfaction), while the role of macro-organizational characteristics (such as ownership and bed size) is relatively weak, which is consistent with the univariate analysis results of this study. Second, face culture, high-power distance, other Chinese-specific cultural factors revealed by the interviews, and phenomena such as organizational silence and a lack of psychological safety are important sources of residual variation in quantitative models. Although these factors are difficult to directly quantify, they are associated with PSC formation and employee behavior, highlighting the value of mixed-methods approaches to capture complex associations.

### 4.6 Study limitations

Although this study made several valuable findings, caution is warranted when interpreting and generalizing the results due to various limitations. First, the quantitative research phase employed a convenience sampling method, with study participants primarily concentrated in South China. Although convenience sampling is widely used and cost-effective, it’s geographical, demographic, and cultural representativeness is limited, which may restrict the applicability of the findings to nursing homes in other regions. Future research could address this limitation by employing more rigorous sampling strategies, such as multi-stage random sampling, to enhance the generalizability and external validity of the findings.

Second, as this study employed a cross-sectional design to explore factors associated with PSC, we cannot draw definitive causal inferences. Although our qualitative findings provide in-depth explanations of possible explanatory pathways, future research should adopt a longitudinal design to track the dynamic changes in PSC and assess the long-term effects of different interventions on PSC and related patient safety outcomes (43).

Third, the qualitative research phase involved focus group interviews. Although maximum variation purposive sampling was employed to ensure sample diversity, and efforts were made to reduce bias by establishing trust and emphasizing anonymity, the findings may still be subject to social desirability bias. This bias refers to the tendency of respondents to provide answers that align with social expectations or organizational norms rather than their true thoughts (31, 44). Despite the researchers’ best efforts to minimize this, its potential impact cannot be overlooked. Additionally, the interviewer visited nursing homes as a training instructor and collaborative researcher prior to conducting interviews, which may have introduced social desirability bias and role-related influence. The participants may have viewed the interviewer as an expert or evaluator, especially in hierarchical institutions. Steps taken to reduce this influence included emphasizing anonymity, establishing rapport, and using neutral questioning techniques, but potential bias remains a limitation.

Fourth, the quantitative research phase primarily focused on the perspectives of frontline staff, with caregivers constituting 84.1% (*n* = 567) of the sample, while RNs represented only 6.7% (*n* = 45) and physicians 3.1% (*n* = 21). In the qualitative phase, although 11 managers were interviewed, their number was small relative to caregivers, and policymakers were not included. Given the critical role of nursing home administrators and policymakers in the construction of PSC, the limited representation of their perspectives may restrict a thorough understanding of organizational-level factors associated with PSC (45). Future mixed-methods research should more explicitly integrate the perspectives of management and policymakers as core stakeholders; for example, through dedicated interviews or surveys, to gain a more comprehensive understanding of their decision-making processes and insight into PSC development and intervention strategies.

Fifth, the study surveyed 674 staff within 11 nursing homes using convenience and cluster sampling approaches. The regression analysis treated all staff members as independent observations without accounting for clustering by nursing home. This may be inappropriate because staff responses may be correlated due to shared leadership, staffing patterns, institutional culture, ownership, policies, and reporting systems. If clustering is ignored, standard errors may be underestimated, and *p*-values may be overly optimistic. We conducted a sensitivity analysis by running the regression model with and without facility-level dummy variables. The pattern of significant predictors remained consistent, and coefficients changed by less than 10% (see Section 2.5.1). However, cluster-robust standard errors were not computed, and this sensitivity check could not fully substitute for multilevel modeling. Future research should address clustering through multilevel modeling, generalized estimating equations, or cluster-robust standard errors.

Additionally, 98.4% of the participants reported working more than 40 h per week, indicating extreme imbalance in working hours that limits the usefulness of this variable as a predictor. Future research should consider more precise workload variables.

Furthermore, the 5.3% reporting rate was derived from the administrative records of a single nursing home; comparable records from the other 10 facilities were unavailable. Records were not kept using a standardized taxonomy, their completeness could not be independently verified, and events never brought to management’s attention could not be captured. This rate therefore reflects formally documented reporting behavior in one facility rather than the true incidence of adverse events across all 11.

Finally, this study is a secondary analysis of existing quantitative and qualitative data from a research program; the data were not originally collected with PSC as the primary outcome, which may constrain the depth of the available measures and interview content for this specific research question.

### 4.7 Future research directions

Based on the findings of this study and the identified limitations, we suggest the following directions for future research to further deepen the understanding of PSC in nursing homes and to promote improvements in practice.

First, future research should comprehensively explore the complex interplay of individual, organizational, and sociocultural factors associated with PSC. This includes developing and validating targeted interventions for identified weak dimensions, such as Staffing and Adherence to Workflow. Scenario-based interprofessional team training (42) could be used to enhance staff collaboration and workflow adherence under conditions of workforce shortages. Concurrently, it is crucial to investigate effective strategies to mitigate organizational silence, which is a significant contributor to low adverse event reporting rates. This could involve integrating aspects of traditional Eastern wisdom into leadership training to foster managers’ openness and supportiveness toward employee feedback (14), along with establishing non-punitive and anonymous reporting mechanisms (46). Studies are also needed to examine how the adverse associations of face culture and high-power distance with reporting behavior can be mitigated, such as by advocating for a collective learning culture of acknowledging mistakes without punishment. Furthermore, future PSC models should be optimized by incorporating variables that accurately describe work characteristics (e.g., shift burden, night shift ratio, and workflow complexity) (45). It is also necessary to explore the association between perceived organizational support and psychological safety (22) to enhance the models’ explanatory power.

Second, longitudinal studies are needed to track dynamic changes in PSC and assess the long-term effects of various interventions on PSC and related patient safety outcomes (43).

Third, the research scope should be expanded to compare differences in PSC across diverse nursing home contexts (e.g., in different regions of China or across government-operated and privately-operated, and integrated medical-nursing facilities). It is also vital to incorporate the perspectives of additional stakeholders, including residents’ families and policymakers, to gain a more comprehensive and multidimensional understanding of nursing home PSC (16) and explore the underlying policy, economic, and cultural factors associated with PSC to inform national-level policy formulation.

## 5 Conclusion

This study employed a mixed-methods approach to systematically assess PSC among nursing homes in South China. Despite an overall high level of PSC, our findings identified significant challenges, such as workforce shortages, occupational burnout, non-standardized work processes, interprofessional role conflicts, and organizational silence associated with Chinese cultural contexts (e.g., face culture, doctrine of the mean) and social-psychological factors (e.g., lack of psychological safety) (45, 47). By exploring the interplay between culturally specific factors such as face culture (mianzi), high-power distance, and family interference, as well as organizational management deficiencies, our findings suggest possible explanatory pathways for the barriers to adverse event reporting observed in one facility’s records. Based on these findings, we suggest potential employee-centered directions for improvement that emphasize organizational psychological safety, interprofessional collaboration, and systematic management reforms.

At the theoretical level, this study explores how PSC may be understood in consideration of the elements of Chinese culture, offering a contextually informed perspective on PSC theory in non-Western settings. Specifically, we describe surface compliance and deep silence as a contextual interpretation emerging from the qualitative data, which may complement psychological safety theory in cultural sensitivity research. At the practical level, the mixed-methods approach used in this study offers a framework for generating hypotheses about patient safety improvement priorities, informed predominantly by the perspectives of caregivers, who comprised 84.1% of the quantitative sample. These potential priorities include optimizing human resource allocation (34), enhancing procedural clarity and reducing interprofessional role conflicts (31), developing non-punitive reporting mechanisms to encourage transparent adverse event reporting (46), and cultivating culturally sensitive leadership that promotes open dialog and feedback (14, 32). Since the quantitative phase employed a cross-sectional design, causal inferences could not be drawn. Future longitudinal or intervention studies are needed to test these hypotheses. Overall, these findings contribute to the empirical understanding of PSC in Chinese nursing homes and may inform future research and intervention development aimed at addressing challenges associated with an aging population.

## Data Availability

The datasets presented in this article are not readily available because the datasets generated and/or analyzed during the current study are not publicly available due to confidentiality agreement with participating nursing homes. Requests to access the datasets should be directed to Zhangan Wang, nnwza@qq.com.
