# Supplementary material for: Patient safety culture in Chinese nursing homes: a mixed-methods study
Source: Front Public Health. 2026 Sep 9;14:1821526. doi: 10.3389/fpubh.2026.1821526 (PMC13597744; doi:10.3389/fpubh.2026.1821526)
Supplement: Supplementary file 1 [file Supplementary_file_1.docx]

**Supplementary Material 1. Joint Display: Integrating Quantitative and Qualitative Findings on Patient Safety Culture in Chinese Nursing Homes**

This joint display links the quantitative findings (the two weak PSC dimensions and the reporting paradox) to the qualitative themes, illustrative quotations, integrated interpretations, and practice implications described in Sections 2.5.3 and 3.3 of the manuscript.

| **Quantitative Finding** | **Qualitative Theme (Manuscript Section 3.3.1)** | **Illustrative Quotations (Participant ID)** | **Integration Pattern** | **Integrated Interpretation** | **Practice Implication** |
| --- | --- | --- | --- | --- | --- |
| Staffing: PRR 67.0%, mean 3.64 ± 0.62 — the weakest dimension (Table 2). The staff-turnover item was rated lowest of all 23 items (PRR 48.5%, mean 2.89 ± 1.21). | Theme 1: Safety Anxiety Associated with Workforce Shortages | “During the day shift, there is only 1 RN and 3 caregivers to care for 45 disabled older individuals, and it is truly impossible to turn them every 2 hours.” (C FG1-02) / “At 3 AM, an older resident fell while using the restroom. Another caregiver went to call the on-duty doctor, and at the same time, multiple residents were calling for assistance. I was running between several residents, and in that moment, I just wanted to cry.” (C FG2-05) | Convergent (explaining) | Qualitative accounts directly substantiate the low Staffing PRR: chronic caregiver-to-resident overload, especially at night, makes protocol-compliant care physically unattainable and normalizes safety anxiety. The near-universal prevalence of working more than 40 hours per week (98.4%, Table 1) further corroborates normalized understaffing (Section 4.1). | Optimizing caregiver-to-resident ratios and strengthening staff coping capacity through simulation training are candidate priorities for intervention development; their effectiveness remains to be tested (Section 4.7). |
| Adherence to Workflow: PRR 72.7%, mean 3.74 ± 1.10 — the second-weakest dimension (Table 2). | Theme 2: Non-Standardized Workflows and Interprofessional Role Conflicts | “The SOP outlines 17 steps for oral care, but no one has demonstrated it. We relied on our own exploration and ultimately simplified it to three steps.” (C FG1-03) / “The physician’s orders are to 'assess the skin and turn the patient every 2 hours', but the night shift nurse believes that is the aide's responsibility, while the aide states 'I cannot assess the grading of pressure injuries.'” (N FG3-02) / “3–4 people leave every month, and new hires are trained for only 3 days before they directly take over shifts, yet many remain unfamiliar with workflows.” (C FG1-01) | Convergent (explaining + expanding) | The intermediate PRR masks a gap between written procedures and enacted practice: procedures that are never demonstrated are informally simplified, role boundaries among physicians, RNs, and caregivers are contested, and high turnover prevents workflow knowledge from consolidating. | Workflow standardization with demonstrated training and interprofessional scenario simulation may improve adherence; these participant-suggested strategies await empirical testing (Theme 4). |
| Reporting paradox: overall PSC PRR 86.5% (mean 4.08 ± 0.42) versus a formally documented adverse event reporting rate of 5.3% from one government-operated nursing home's administrative records (25 of 476 frontline staff; Section 2.3.1). | Theme 3: Cultural Barriers to Adverse Event Reporting | “Last time when an older resident fell, the nursing department responded with 'Why weren't you more careful?' providing only criticism without any guidance or assistance. Since then, everyone believes that 'reporting is useless,' and no one in the department is willing to report anymore.” (N FG3-03) / “Admitting to giving the wrong medication in the morning meeting? People will think your skills are poor, and your chances for promotion will definitely be jeopardized.” (N FG3-02) / “For any reason, every time an older resident falls, the on-duty staff is fined 100 RMB ($13.9), which affects the overall group's performance.” (N FG3-02) / “The first thing the family said was, 'You must compensate us.' Therefore, for events that are not obvious, there is a general tendency to handle them privately and not report them to the system.” (C FG1-03) | Complementary (dissonance resolved) | Qualitative data resolve the surface contradiction: survey PRR captures espoused perceptions, whereas reporting behavior is governed by blame-oriented feedback, face culture, punitive fines, and compensation risks. This pattern is described in the manuscript as surface compliance and deep silence — positive survey responses coexisting with withheld incident reports in a high-power-distance, face-culture context (Sections 4.3 and 5). | Non-punitive reporting channels with structured feedback loops are the highest-priority hypothesis emerging from the integrated findings (Sections 4.4 and 4.7). |
| Improvement directions derived from both phases. | Theme 4: Staff-driven Strategies for Improving Patient Safety | “We can learn from the adverse events reported by others (a lesson learned from a setback).” (M FG5-05) / “It is recommended to hold a 'near-miss' sharing session once a month, without assigning blame, focusing solely on how to mitigate risks.” (M FG6-01) / “By having doctors, RNs, and caregivers simulate emergency situations during night shifts, they come to understand each other's challenges.” (N FG4-01) | Expanding | Frontline staff themselves proposed the two strategies most consistent with the integrated diagnosis: non-punitive reflective learning spaces (targeting Theme 3 barriers) and interprofessional scenario simulation training (targeting Themes 1–2 weaknesses). | Both strategies are framed as hypotheses or priorities for future intervention development and evaluation, not as proven solutions (Section 4.7). |

*Notes. PRR = positive response rate (percentage of “agree” or “strongly agree” responses for positively worded items, and of "disagree" or "strongly disagree" responses for the three reverse-coded items; see Section 2.3.1). Participant IDs: C = caregiver, N = registered nurse (RN), M = manager; FG = focus group; the first digit is the focus-group sequence number and the second is the anonymized respondent number, consistent with Section 3.3 of the manuscript. Quotations were translated from Mandarin Chinese by the first author and independently back-translated by the corresponding author. Quantitative data were collected between August 2021 and January 2022; focus group interviews were conducted between May and June 2022. The connection between the two datasets occurred at the analysis and interpretation stage, as described in Section 2.5.3 of the manuscript.*

*Design rationale. This joint display follows the integration-through-narrative and joint-display guidance of Fetters, Curry, and Creswell (2013) for mixed-methods integration at the interpretation stage, and the connecting, explaining, and expanding strategies described in Section 2.5.3 of the manuscript. Each row juxtaposes a quantitative pattern with the qualitative evidence that explains or expands it, so that convergence and dissonance are inspectable rather than asserted.*

*Reference for joint display methodology. Fetters MD, Curry LA, Creswell JW. Achieving integration in mixed methods designs—principles and practices. Health Serv Res. (2013) 48:2134–56. doi: 10.1111/1475-6773.12117*
