# Supplementary material for: Patient safety culture in Chinese nursing homes: a mixed-methods study
Source: Front Public Health. 2026 Sep 9;14:1821526. doi: 10.3389/fpubh.2026.1821526 (PMC13597744; doi:10.3389/fpubh.2026.1821526)
Supplement: Supplementary file 2 [file Supplementary_file_2.docx]

**Supplementary Material 2. Reporting Checklists (STROBE and COREQ)**

All locations refer to the revised manuscript. Items not applicable to the study design are marked N/A with a brief reason; items that are only partially addressed are marked Partial with an explanation.

**PART A — STROBE Checklist (Cross-Sectional Studies), Quantitative Phase**

| **Item** | **Recommendation** | **Reported** | **Location / Note** |
| --- | --- | --- | --- |
| 1a | Indicate the study's design with a commonly used term in the title or abstract | ✓ | Title: “Patient Safety Culture in Chinese Nursing Homes: A Mixed-Methods Study”; Abstract, Design: “A mixed-methods study based on a secondary analysis of a multisite research program, integrating a 2021–2022 cross-sectional survey with a secondary thematic analysis of focus group interviews” |
| 1b | Informative and balanced structured abstract | ✓ | Abstract (275 words): Objective, Design, Setting and Participants, Methods, Results, Conclusions and Implications |
| 2 | Scientific background and rationale | ✓ | Introduction, paragraphs 1–5 |
| 3 | Specific objectives / hypotheses | ✓ | Introduction, final two paragraphs: research aims covering PSC status, associated factors, and possible explanatory pathways |
| 4 | Key elements of study design early in the paper | ✓ | Section 2.1: mixed-methods design; quantitative phase = 2021–2022 cross-sectional survey; qualitative phase = secondary thematic analysis of the focus-group corpus |
| 5 | Setting, locations, relevant dates | ✓ | Sections 2.1.1, 2.2.1, 2.4.1: 11 nursing homes in South China; survey conducted between August 2021 and January 2022 |
| 6a | Eligibility criteria; sources and methods of participant selection | ✓ | Section 2.2.1: frontline staff with ≥ 1 year of experience; exclusion criteria specified; convenience sampling with a cluster approach |
| 6b | Matched studies: matching criteria | N/A | Not a matched study |
| 7 | Define outcomes, exposures, predictors, confounders | ✓ | Sections 2.3.1, 2.5.1: outcome = total PSC score (mean of the 23 NHSOPSC items); candidate predictors pre-specified (facility operation mode, facility size, years of work experience, income satisfaction, job satisfaction) |
| 8 | Sources of data and measurement details | ✓ | Section 2.3.1: Chinese version of the NHSOPSC, 23 items, 6 dimensions, 5-point Likert scale, reverse coding; reliability reported for the prior validation and the present sample, the 5.3% reporting rate derived from one facility's administrative records (Section 2.3.1) |
| 9 | Efforts to address potential sources of bias | ✓ | Sections 2.2.1 (broad facility representation), 2.4.1 (independent review and double verification of electronic and paper questionnaires), 2.5.1 (pre-specified predictors, collinearity checks, sensitivity analysis) |
| 10 | How the study size was arrived at | ✓ | Section 2.1.1: We conducted an a priori power analysis using G*Power software (linear multiple regression: fixed model, R² deviation from zero) (19) with 80% statistical power (power = 0.80), a 0.05 significance level (α = 0.05), five predictors in the multiple linear regression model, and a small expected effect size (f² = 0.02); the estimated minimum required sample size was 647 cases. The final analytic sample (n = 674) met this requirement. |
| 11 | How quantitative variables were handled | ✓ | Sections 2.3.1, 2.5.1: PRR computation; work experience dichotomized as 1–3 versus ≥ 3 years uniformly across descriptive, univariate, and regression analyses; five-level satisfaction variables collapsed into three categories five-level satisfaction variables collapsed into three categories |
| 12a | All statistical methods, including control of confounding | ✓ | Section 2.5.1: t-tests/ANOVA (Levene’s test, Welch’s ANOVA, Bonferroni adjustment), multiple linear regression with simultaneous entry of univariately significant predictors; model diagnostics (F, R², adjusted R², Shapiro–Wilk, Breusch–Pagan) |
| 12b | Subgroup and interaction methods | N/A | No formal subgroup or interaction analyses; role-stratified analysis not conducted due to insufficient non-caregiver sample sizes (Section 2.2.1) |
| 12c | How missing data were addressed | Partial | Section 2.4.1: analysis restricted to the subsample with complete and valid NHSOPSC data (674 of 750); item-level quality-control rules are reported in the parent-study publication (reference 10) |
| 12d | Analytical methods taking account of sampling strategy | Partial | Sections 2.5.1 and 4.6: nesting within 11 nursing homes acknowledged; facility-dummy sensitivity analysis reported; multilevel modeling not used because the number of clusters (n = 11) was insufficient |
| 12e | Sensitivity analyses | ✓ | Section 2.5.1: regression with versus without facility-level dummy variables; coefficients changed by less than 10% |
| 13a | Numbers at each stage of the study | ✓ | Section 2.4.1 and Figure 1: 940 staff surveyed → 750 completed questionnaires → 674 with complete and valid NHSOPSC data |
| 13b | Reasons for non-participation at each stage | ✓ | Section 2.4.1: < 1 year of experience or long-term absence (n = 125); declined to participate (n = 65) |
| 13c | Flow diagram | ✓ | Figure 1 (flow diagram of quantitative participant recruitment) |
| 14a | Characteristics of study participants | ✓ | Table 1 (n = 674) |
| 14b | Participants with missing data per variable | Partial | Exclusion-based handling reported (Section 2.4.1); per-variable missingness not itemized |
| 14c | Follow-up time (cohort) | N/A | Cross-sectional design |
| 15a | Outcome/exposure descriptive data | ✓ | Table 2 (dimension PRR and scores); Table 3 (univariate analyses) |
| 16 | Outcome events / summary measures | ✓ | Tables 2–4 |
| 17a | Unadjusted and adjusted estimates with precision | ✓ | Table 4: B, SE, β, 95% CI, t, and p reported for every predictor |
| 17b | Which confounders were adjusted for and why | ✓ | Section 2.5.1: predictors pre-specified from theory and prior literature; univariable screening at p < 0.05; reference categories specified |
| 17c | Relative risk translation | N/A | Continuous outcome analyzed by linear regression |
| 18 | Other analyses (subgroups, sensitivity) | ✓ | Facility-dummy sensitivity analysis (Sections 2.5.1 and 4.6) |
| 19 | Key results summarized with reference to objectives | ✓ | Discussion, Sections 4.1–4.3 |
| 20 | Limitations, sources of bias/imprecision | ✓ | Section 4.6: convenience sampling, cross-sectional design, social desirability bias, interviewer dual role, caregiver-predominant sample, unmodeled clustering, constraints of secondary analysis |
| 21 | Cautious overall interpretation | ✓ | Non-causal language throughout; Sections 2.5.1, 4.6, and 5 |
| 22 | Generalizability | ✓ | Sections 4.1 and 4.6 (South China focus; caregiver-predominant sample) |
| 23 | Funding | ✓ | Funding statement: Guangxi Project for the Development and Promotion of Appropriate Medical and Health Technologies, China (grant No. S2023022) |

**PART B — COREQ Checklist (32 Items), Qualitative Phase**

| **No.** | **Item** | **Reported** | **Location / Note** |
| --- | --- | --- | --- |
| **Domain 1: Research team and reflexivity** | | | |
| 1 | Interviewer/facilitator identified | ✓ | Section 2.4.2: interviews facilitated by an associate professor with extensive experience in qualitative research, assisted by an observer who took notes |
| 2 | Credentials | ✓ | Section 2.4.2 (associate professor, extensive qualitative research experience) |
| 3 | Gender of interviewer | Not reported | Not stated in the manuscript |
| 4 | Experience and training | ✓ | Section 2.4.2 |
| 5 | Relationship with participants established | ✓ | Section 2.4.2: pre-observation period; the interviewer visited the nursing homes as a training instructor and collaborative researcher to build rapport and mutual trust |
| 6 | Participant knowledge of the interviewer | ✓ | Section 2.4.2: independent-researcher status declared at the start of each interview; the dual role as training instructor was explicitly acknowledged and mitigated (steps a–d) |
| 7 | Interviewer characteristics / positionality | ✓ | Section 2.4.2: mitigation steps (a)–(d) reported; the researcher documented observations and personal feelings throughout data collection and transcription to enhance reflexivity |
| **Domain 2: Study design** | | | |
| 8 | Methodological orientation and theory | ✓ | Sections 2.1.2, 2.5.2: thematic analysis per Braun and Clarke (reference 28) within a mixed-methods design (secondary analysis oriented by the quantitative findings) |
| 9 | Sampling | ✓ | Section 2.2.2: maximum variation purposive sampling across roles, years of service, and facility ownership types |
| 10 | Method of approach | ✓ | Section 2.2.2: recruitment posters within the nursing homes and face-to-face briefings by researchers |
| 11 | Sample size | ✓ | Sections 2.2.2, 3.1.2: 6 focus groups, n = 35 (12 caregivers, 12 RNs, 11 managers), 5–6 participants per group |
| 12 | Non-participation | Not reported | Numbers of and reasons for declining qualitative participation were not recorded |
| 13 | Setting of data collection | ✓ | Sections 2.2.2, 3.1.2: on-site at the two participating nursing homes (one government-operated and one privately-operated) |
| 14 | Presence of non-participants | ✓ | Sections 2.2.2, 3.1.2: managers were not present during caregiver or RN interviews; role-separated groups |
| 15 | Description of sample | ✓ | Section 3.1.2 and Supplementary Table 1 (role, gender, age range, facility ownership, interview duration) |
| 16 | Interview guide | ✓ | Section 2.3.2: guide developed in consultation with two senior nursing management specialists and a qualitative research expert; pilot tested; five core questions listed |
| 17 | Repeat interviews | N/A | None conducted |
| 18 | Audio/visual recording | ✓ | Section 2.4.2: audio recorded by the moderator and the observer; transcribed verbatim within 24 hours |
| 19 | Field notes | ✓ | Section 2.4.2: observer field notes; nonverbal cues recorded; reflexive documentation |
| 20 | Data duration | ✓ | Sections 2.4.2, 3.1.2: interviews conducted between May and June 2022; 38–62 minutes per group (mean = 51 minutes) |
| 21 | Data saturation | ✓ | Sections 2.2.2, 2.4.2: no new themes or perspectives across two consecutive focus groups |
| 22 | Transcripts returned to participants | ✓ (negative reported) | Section 2.4.2: full transcripts not returned (reasons given); key themes verified with participants at the end of each interview |
| **Domain 3: Analysis and findings** | | | |
| 23 | Number of data coders | ✓ | Section 2.5.2: two independently trained researchers; consensus meetings including a third expert when needed |
| 24 | Description of the coding tree | ✓ | Sections 2.5.2, 3.3: iterative six-phase framework; 4 core themes and 12 sub-themes derived |
| 25 | Derivation of themes | ✓ | Section 2.5.2: Braun and Clarke’s six phases; regular peer debriefing sessions among the three researchers |
| 26 | Software | ✓ | Section 2.5.2: NVivo 14 |
| 27 | Participant checking | ✓ | Section 2.4.2: key themes verified with participants at the end of each interview; full transcripts not returned (stated) |
| 28 | Quotations presented | ✓ | Section 3.3.1: quotations with anonymized participant IDs (role + focus-group number) |
| 29 | Data and interpretation consistency | ✓ | Themes mapped to the quantitative weak dimensions (Sections 2.5.3, 3.3.1) and the joint display (Supplementary Material 1) |
| 30 | Clarity of major themes | ✓ | Four themes with definitions and theme–subtheme–evidence chains (Section 3.3.1) |
| 31 | Clarity of minor themes | ✓ | Twelve sub-themes presented under the four major themes (Section 3.3.1) |
| 32 | Inter-coder reliability | ✓ | Section 2.5.2: Cohen’s kappa = 0.84, indicating almost perfect agreement |

*Note. COREQ items 3 and 12 are the only elements not addressed in the revised manuscript; both are reporting gaps rather than methodological omissions. NHSOPSC = Nursing Home Survey on Patient Safety Culture; PRR = positive response rate; RN = registered nurse; STROBE = Strengthening the Reporting of Observational Studies in Epidemiology; COREQ = Consolidated Criteria for Reporting Qualitative Research.*
