# Supplementary material for: Patient safety culture in Chinese nursing homes: a mixed-methods study
Source: Front Public Health. 2026 Sep 9;14:1821526. doi: 10.3389/fpubh.2026.1821526 (PMC13597744; doi:10.3389/fpubh.2026.1821526)
Supplement: Supplementary file 3 [file Supplementary_file_3.docx]

**Supplementary Table 1. Characteristics of Qualitative Focus Group Participants (n = 35)**

| **Characteristic** | **Category** | **Detail** |
| --- | --- | --- |
| **Job role** |  |  |
|  | Caregivers | 2 focus groups, n = 12 (34.3%) |
|  | RNs | 2 focus groups, n = 12 (34.3%) |
|  | Managers | 2 focus groups, n = 11 (31.4%) |
| **Gender** |  |  |
|  | Male | n = 4 (11.4%) |
|  | Female | n = 31 (88.6%) |
| **Educational level** |  |  |
|  | Caregivers: primary school | 9 (75.0) |
|  | Caregivers: junior high school | 3 (25.0) |
|  | Nurses: technical secondary school | 10 (83.3) |
|  | Nurses: associate degree | 2 (16.7) |
|  | Managers: associate degree | 7 (63.6) |
|  | Managers: bachelor's degree | 2 (18.2) |
|  | Managers: technical secondary school | 2 (18.2) |
| **Facility operation mode** | |  |
|  | Government-operated | 1 |
|  | Privately-operated | 1 |
| **Age** | M ± SD (range) | 43.6 ± 10.9 (23–61) |
| **Years of experience in nursing home care** | M ± SD (range) | 10.7 ± 10.0 (1–35) |
| **Interview duration** | Range (mean) | 38–62 minutes (mean = 51 minutes) |

*Note: Focus groups were conducted separately by job role (5–6 participants per group). Managers were not present during caregiver or nurse interviews. Interviews were conducted between May and June 2022.*
